# Health reform and mortality in China: Multilevel time-series analysis of regional and socioeconomic inequities in a sample of 73 million

**DOI:** 10.1038/srep15038

**Published:** 2015-10-15

**Authors:** Thomas Astell-Burt, Yunning Liu, Xiaoqi Feng, Peng Yin, Andrew Page, Shiwei Liu, Jiangmei Liu, Lijun Wang, Maigeng Zhou

**Affiliations:** 1 School of Science and Health, Western Sydney University, Sydney, Australia; 2 School of Geography and Geosciences, University of St Andrews, St Andrews, United Kingdom; 3 China Center for Disease Control and Prevention, Beijing, People’s Republic of China; 4 Early Start Research Institute, University of Wollongong, Wollongong, Australia; 5 School of Health and Society, University of Wollongong, Wollongong, Australia; 6 School of Medicine, Western Sydney University, Sydney, Australia

## Abstract

China’s 2009 expansion of universal health insurance has received global interest, but little empirical investigation. This epidemiological study was a first attempt to assess potential impacts on population health and health equity. Multilevel negative binomial regression was used to analyse all-cause and non-communicable disease (NCD) mortality between 2006 and 2012 from a representative sample including all 31 provinces. The age-standardised ratios (per 100,000) in 2006 were 860.4 and 732.9 for mortality from all-causes and NCDs respectively. These ratios decreased over time to 737.5 (all-causes) and 642.9 (NCD) by 2012. Modelling indicated these trajectories were curvilinear, dipping more rapidly from 2009 onwards. Compared to the east, all-cause mortality was higher in other regions (e.g. northwest RR: 1.34, 95% CI: 1.20, 1.48). Compared to more affluent urban areas, rate ratios for all-cause mortality were 1.23 (95% CI: 0.97, 1.54) in the least affluent urban areas, 1.22 (95% CI: 1.02, 1.46) in affluent rural areas and 1.64 (95% CI: 1.51, 1.79) in the least affluent rural areas. These health inequities were largely repeated for NCD mortality and did not vary spatiotemporally. Overall, universal health insurance in China may have accelerated reductions in all-cause and NCD mortality, but potential impacts on health inequity may take longer to manifest.

Universal access to good quality health services helps protect individuals from disease, benefiting socioeconomically disadvantaged groups^1^ and paving the way for a stronger labour force^2^. In 2009, the Chinese government introduced a CN¥850 billion (US$125 billion) national health policy reform package, leading to a substantial increase in access to health insurance to over 90% of the population by the end of 2010^3^. To date, scant large-scale empirical evidence has been accumulated within the scientific literature on the potential impacts these reforms have had on population health and health inequities in China^4^. Accordingly, the purpose of this study was to examine regional and socioeconomic trajectories in all-cause and non-communicable disease (NCD) mortality before and since the 2009 health reforms. The ambition was to assess whether (i) a potential early impact of the health reform could be observed and (ii) if such an impact was confined to specific regions or particular socioeconomic circumstances.

## Method

### Data

The Chinese Center for Disease Control and Prevention (China CDC) is the data custodian for a nationally representative mortality time-series that is collected via 161 ‘Disease Surveillance Points’ (DSP). The DSP system covers a sample population of approximately 73 million people, including urban and rural counties in all 31 provinces, municipalities and autonomous regions within China.

### Outcome variables

All-cause and NCD disease mortality counts were stratified by year, gender and age group (20–34y, 35y–49y, 50–64, 65y and older) at the DSP level for years 2006 to 2012 inclusive. Population counts from the 2010 census were used to ascertain denominators for mortality rates.

### Socioeconomic and urban/rural variables

Socioeconomic circumstances were measured using the mean years of education among residents within each county, extracted from the 2010 census. This measure was expressed in tertiles and further stratified according to whether the DSP was classified as urban (n = 64) or rural (n = 97), to afford an opportunity to differentiate between levels of socioeconomic circumstances within urban/rural categories. Additionally, all DSPs were classified in a separate variable according to their regional affiliation.

### Statistical analysis

Descriptive analysis of mortality trajectories across time were implemented using age-adjusted standardised ratios. Further analysis of each mortality count was implemented using negative binomial random effects (‘multilevel’) regression^5^ to account for over-dispersion and the hierarchical data structure, with the natural logarithm of equivalently stratified total population counts extracted from the census (adjusted for annual change) fitted as the offset. Initial models were fitted with dummy variables denoting gender, age group and year of observation. Dummy variables for each year were preferred over linear and quadratic terms as the latter tends to have a smoothing effect on the period variable, making it difficult to detect potentially sharp changes in the outcome variables. The multilevel models assessed the degree of geographical variation in each outcome using the Median Rate Ratio (MRR) method^6^. Dummy variables for the socioeconomic circumstances and region variables were added as fixed effect parameters. In order to explore for spatiotemporal variation, interaction terms for year by socioeconomic circumstances and year by region were fitted. This analytical strategy was conducted for each mortality outcome separately. All fixed effect parameters were exponentiated to rate ratios (RR) and 95% confidence intervals (95% CI). Predicted probabilities and 95% CIs were used to visualise mortality trajectories. All analyses were conducted in MLwIN v2.30.

## Results

The age-standardised ratios (per 100,000) in 2006 were 860.4 and 732.9 for mortality from all-causes and NCDs respectively. These ratios decreased over time to 737.5 (all-causes) and 642.9 (NCD) by 2012. Between 2006 and 2008 inclusive, all-cause and NCD mortality trajectories appeared relatively consistent over time. From 2009 onwards, however, there were notable decreases in both outcomes. Caterpillar plots of the area-level residuals (expressed as rate ratios and 95% CIs) showed substantial geographical variation for each mortality outcome. MRRs estimated from random intercept models adjusted for age, gender and year were 1.35 for all-cause mortality and 1.30 for NCD mortality.

**Figure 1:**
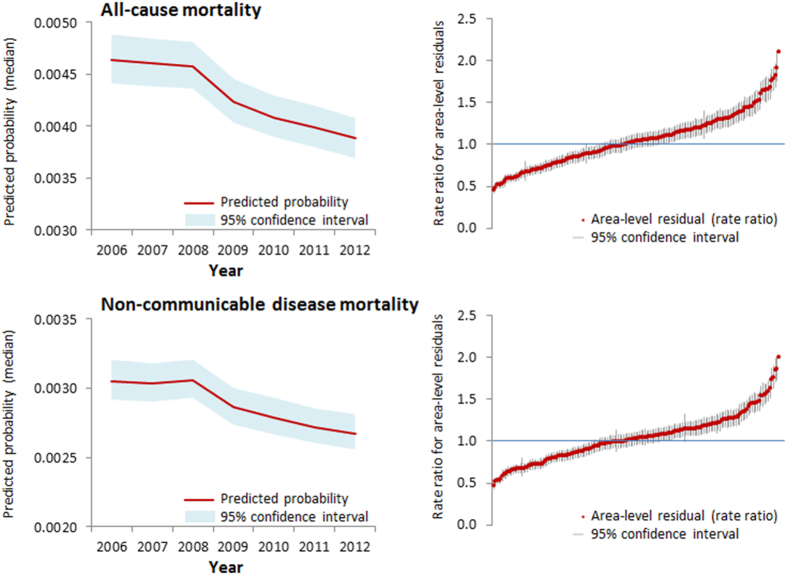
Predicted probabilities and area-level residuals for all-cause and non-communicable disease mortality, estimated from multilevel negative binomial regression.

The multilevel models used to construct Fig. 1 are shown in Table 1 (Model 1). Females had a lower rate ratio than their male counterparts of the same age across both mortality outcomes. The non-linear trajectories in each outcome over time were also in evidence, with steeper declines in rate ratios post-2009.

Region and the cross-classification between urban/rural and county-level mean years of education accounted for 63% and 55% of the geographic variation in all-cause and NCD mortality outcomes respectively. There was weak statistical evidence for interaction terms with year (*p*>0.05), indicating that the geographic variations were consistent over time (i.e. no region-specific trajectories or deviations for a particular urban/rural or education year category).

Compared to the east region (reference category), all-cause mortality was consistently higher in all other regions, especially in the southwest (RR: 1.30, 95% CI: 1.17, 1.44) and northwest (RR: 1.34, 95% CI: 1.20, 1.48). NCD mortality was also higher in other regions compared to the east, particularly in the northwest (RR 1.29, 95% CI: 1.16, 1.43) and the northeast (RR 1.30 (95% CI: 1.16, 1.45).

All-cause and NCD mortality tended to be lower in urban than rural areas, with evidence of effect measure modification by area-level socioeconomic circumstances. Compared to more affluent urban areas, rate ratios for all-cause mortality were 1.23 (95% CI: 0.97, 1.54) in the least affluent urban areas, 1.22 (95% CI: 1.02, 1.46) in affluent rural areas and 1.64 (95% CI: 1.51, 1.79) in the least affluent rural areas. This pattern was largely repeated for NCD mortality, with rate ratios in comparison to affluent urban areas of 1.15 (95% CI: 0.91, 1.44) in the least affluent urban areas, 1.18 (95% CI: 0.98, 1.42) in affluent rural areas and 1.53 (95% CI: 1.41, 1.66) in the least affluent rural areas. No spatiotemporal variations in mortality by these area-level variables were observed.

## Discussion

To the authors knowledge, this is the first large-scale longitudinal analysis using nationally representative data to observe potential impacts of the 2009 health reform package on population health and health equity in China. Overall, this study suggests that the rapid expansion of (near) universal health insurance across China may have had a positive impact on population health. Impacts on health inequities are yet to emerge. The significance of this information for health policy makers is contextualised in the unprecedented change in built, physical and socioeconomic environments associated with rapid urbanisation has evolved unevenly across China. Health reform over the past 10–15 years, though especially since 2009 in the dramatic expansion of health insurance to over 90% of the population, has been a key government response. The long-term impact of the reforms on population health will not be known for some time, however, the present study suggests that all-cause and NCD mortality has decreased in China at a faster rate since the health reform period 2009 onwards. While there does not appear to have been a narrowing of socioeconomic or regional differences in the mortality indicators analysed, importantly, there is no evidence for widening inequities either. The findings therefore support widespread views on the provision of universal health insurance as a fundamental social determinant of health^7^.

### Strengths and limitations

The large sample spanning 161 DSPs across all 31 provinces is a strength of our study, which is enhanced further by the multilevel framework for explicitly analysing variance in mortality across these areas and over time. One limitation relates to criticism in the early appraisals of the 2009 health reform package, including ongoing non-trivial healthcare expenses^8^ and a maldistribution of skilled health workforce^3^. A limitation of this study is that the aforementioned circumstances are also unlikely to be geographically random and, as such, present unmeasured variation in the impact of the expansion in health insurance that could not be incorporated into analyses due to an absence of this data. Both factors may have had an impact on change in demand or supply of particular types of healthcare in some areas. While there did not appear to be any evidence for this in the analyses reported in this study, those variations may matter more for particular health outcomes. Future research would benefit from incorporating detailed information on healthcare supply for relevant geographical units of analysis in order to assess the change in provision of local health services before and after the health reform. Another limitation was the focus on all-cause and NCD mortality, for which we did not observe spatiotemporal variations, but there may well be modification of inequities (widening and narrowing) in particular causes of mortality and, especially, those determined to be ‘amenable’ to health sector reform^10^. Further research to compare trajectories in amenable mortality to causes of death considered less likely to be influenced by universal health coverage (e.g. pancreatic cancer) are warranted in order to strengthen the level of causal inference.

### Interpretation

The potential impacts of rapid urbanisation and economic development on health, health-related behaviours, and health inequities in China have been and continue to be the subject of international public health interest^9^. The geographically uneven distributions of health-relevant exposures such as outdoor and indoor air pollution, and lifestyle risk factors will require multi-sector initiatives championed by the health sector. Uniform improvements in each of these outcomes and exposures (among others) are not inevitable as a result of the expansion in health insurance. Epidemiological studies in China must, by definition, pay close attention to the multilevel nature of exposure-outcome relations and not just overall trends, where localised, but important deviations may be obscured from policy makers and not acted upon in the interests of health equity. It may also be that many of the important impacts of the health reform package for population health and health equity are yet to manifest. Thus, further research that examines particular causes of death and tracks outcomes over longer periods of time is necessary in order to sustain an evidence-based health policy discussion in China.

## Additional Information

**How to cite this article**: Astell-Burt, T. *et al.* Health reform and mortality in China: Multilevel time-series analysis of regional and socioeconomic inequities in a sample of 73 million. *Sci. Rep.* **5**, 15038; doi: 10.1038/srep15038 (2015).

## Figures and Tables

**Table 1:** Regional and socioeconomic differences in all-cause and non-communicable disease mortality in China. Fixed and random effects estimated from multilevel negative binomial regression.

	All-cause mortality		Non-communicable disease mortality	
	Model 1	Model 2	Model 1	Model 2
Fixed Effects	Rate Ratio (95% Confidence Interval)			
Gender & Age Group (ref: Male 20-34y)	1	1	1	1
Female 20–34y	0.45 (0.44, 0.46)	0.45 (0.44, 0.46)	0.64 (0.62, 0.66)	0.64 (0.62, 0.66)
Male 35–49y	3.50 (3.43, 3.58)	3.50 (3.43, 3.58)	6.78 (6.64, 6.93)	6.78 (6.64, 6.93)
Female 35–49y	1.56 (1.53, 1.59)	1.56 (1.53, 1.59)	3.55 (3.47, 3.63)	3.55 (3.47, 3.63)
Male 50–64y	11.51 (11.28, 11.74)	11.51 (11.26, 11.76)	30.02 (29.38, 30.68)	30.02 (29.38, 30.68)
Female 50–64y	6.06 (5.93, 6.19)	6.06 (5.93, 6.19)	16.48 (16.13, 16.84)	16.48 (16.13, 16.84)
Male 65y+	65.63 (64.35, 66.93)	65.69 (64.42, 66.99)	182.00 (178.12, 185.97)	182.00 (178.12, 185.97)
Female 65y+	50.20 (49.22, 51.19)	50.20 (49.22, 51.19)	137.14 (134.21, 140.13)	137.14 (134.21, 140.13)
Year (ref: 2006)	1	1	1	1
2007	0.99 (0.97, 1.01)	0.99 (0.97, 1.01)	1.00 (0.98, 1.01)	1.00 (0.98, 1.01)
2008	0.99 (0.97, 1.01)	0.99 (0.97, 1.01)	1.00 (0.98, 1.02)	1.00 (0.98, 1.02)
2009	0.91 (0.90, 0.93)	0.91 (0.90, 0.93)	0.94 (0.92, 0.96)	0.94 (0.92, 0.96)
2010	0.88 (0.86, 0.90)	0.88 (0.86, 0.90)	0.91 (0.90, 0.93)	0.91 (0.90, 0.93)
2011	0.86 (0.84, 0.88)	0.86 (0.84, 0.88)	0.89 (0.87, 0.91)	0.89 (0.87, 0.91)
2012	0.84 (0.82, 0.85)	0.84 (0.82, 0.85)	0.88 (0.86, 0.89)	0.88 (0.86, 0.89)
Region (ref: east)		1		1
north		1.17 (1.06, 1.30)		1.22 (1.10, 1.35)
central		1.15 (1.04, 1.28)		1.15 (1.04, 1.28)
south		1.17 (1.04, 1.32)		1.19 (1.05, 1.34)
southwest		1.30 (1.17, 1.44)		1.23 (1.11, 1.36)
northwest		1.34 (1.20, 1.48)		1.29 (1.16, 1.43)
northeast		1.19 (1.07, 1.34)		1.30 (1.16, 1.45)
Socioeconomic circumstances		1		1
(ref: Urban, high years of education)				
Urban, moderate years of education		1.27 (1.11, 1.45)		1.24 (1.09, 1.42)
Urban, low years of education		1.23 (0.97, 1.54)		1.15 (0.91, 1.44)
Rural, high years of education		1.22 (1.02, 1.46)		1.18 (0.98, 1.42)
Rural, moderate years of education		1.49 (1.38, 1.62)		1.40 (1.29, 1.52)
Rural, low years of education		1.64 (1.51, 1.79)		1.53 (1.41, 1.66)
Random Effects				
Variance (standard error)	0.097 (0.011)	0.036 (0.004)	0.080 (0.009)	0.036 (0.004)
Median Rate Ratio	1.35	1.20	1.31	1.20
Percentage change in variance	.	62.9%	.	55.0%
